# Survey of pain and stigma experiences in people diagnosed with mpox in Baltimore, Maryland during 2022 global outbreak

**DOI:** 10.1371/journal.pone.0299587

**Published:** 2024-05-21

**Authors:** Sarah Ann Schmalzle, Matthew Grant, Susan Lovelace, Jiwon Jung, Clara Choate, Julie Guerin, Walker Weinstein, Gregory Taylor

**Affiliations:** 1 Institute of Human Virology, University of Maryland School of Medicine, Baltimore, Maryland, United States of America; 2 Department of Medicine, Division of Infectious Disease, University of Maryland School of Medicine, Baltimore, Maryland, United States of America; 3 Department of Pediatrics, University of Maryland School of Medicine, Baltimore, Maryland, United States of America; 4 Goucher College, Baltimore, Maryland, United States of America; 5 University of Maryland Medical Center, Baltimore, Maryland, United States of America; 6 Department of Family Medicine, University of Maryland School of Medicine, Baltimore, Maryland, United States of America; King Faisal University, SAUDI ARABIA

## Abstract

A high prevalence of mpox in men who have sex with men and in people with HIV, plus visually striking and contagious lesions, have raised concerns for mpox stigma. 24 PCR-confirmed mpox patients were surveyed over the course of three months, utilizing an mpox stigma scale adapted from the HIV Stigma Scale plus assessment of pain, analgesic efficacy, and healthcare experiences. Participants were cis-male (100%), with male sexual partners (96%), mostly African-American (88%), and living with HIV (79%). Patients answered 4–16 of 24 (mean 10) stigma questions affirmatively, particularly related to negative effects of mpox on the LGBTQ community. 79% reported pain, most commonly of limbs and perianal area, with perianal pain being rated most severe. The most effective pain relief occurred with opioids (100% major relief, n = 2) and tecovirimat (63% major relief, 25% moderate, n = 16). Patients were satisfied with care provided at the studied clinics, but had negative experiences at all other mentioned sites.

## Introduction

The scale, symptomatology, and demographics of the 2022 mpox (formerly monkeypox) outbreak each diverged from the ‘classic’ outbreaks that have been occurring sporadically in parts of Africa since the first human case was recognized in 1970 [1]. This outbreak has so far spanned 110 countries [2], occurred in patients without a travel link to areas of endemicity [2], included prominent perianal involvement and pain, and appeared to be associated with sexual activity, given its predilection for gay, bisexual, and other men who have sex with men (GBMSM) and co-occurrence with HIV and other sexually transmitted infections (STIs) [3–8].

In anticipation of the potential for a global orthopoxvirus outbreak, the vaccine Jynneos, and the antiviral tecovirimat had been developed in recent years. FDA approval of tecovirimat for smallpox treatment was granted in 2018 and of Jynneos for smallpox and mpox prevention in 2019. Jynneos is an attenuated, live, non-replicating smallpox and mpox vaccine, with efficacy inferred based on immunogenicity and animal challenge studies [9]. Tecovirimat is an orthopoxvirus specific antiviral that prevents formation of egress-competent orthopox virions in the host, which was approved based on lethal challenge studies in non-human primates and rabbits infected with non-variola orthopoxviruses, including mpox [10]. Tecovirimat was made available during the 2022 mpox outbreak through the expanded access investigational new drug process to treat patients with or at risk for severe mpox disease, and patients with involvement of anatomic areas which might result in serious sequelae that include scarring or strictures [11].

Early on, experts compared this outbreak to the beginning of the HIV pandemic and warned of potential for stigma and discrimination due to the groups affected, mode of transmission, and racial and geographical connotations of the official name of the virus and clades. Thus, the WHO changed the clade names from Congo Basin (Central African) clade and West African clade to Clade I and Clade II in August 2022, and the name of the virus from monkeypox virus to mpox virus in November 2022 [12, 13].

There are no published studies examining patient experiences during the 2022 mpox outbreak or analgesic use or efficacy. The lived experiences of people diagnosed with mpox need to be better quantified and understood in order to adjust public health and individual approaches to address this and future outbreaks. Given the parallels with the early years of the HIV epidemic and high rates of mpox occurring in GBMSM living with HIV, the validated HIV stigma scale [14] and abbreviated version of the HIV stigma scale [15] were adapted to a mpox stigma scale. Additionally, experience with pain, pain management modalities, and health care experiences were assessed.

## Methods

### Description of clinical sites

This study was conducted at the Institute of Human Virology THRIVE Program adult HIV clinic, and at the Adolescent and Young Adult Clinic (AYAC), both at the University of Maryland School of Medicine. Both offer HIV prevention and HIV primary care with extensive wrap-around services, and receive Ryan White grant funding. They each offer gender-affirming and culturally competent care to sexual and gender minorities (SGM), and as such became referral sites for mpox evaluation and management including in-person and telemedicine appointments.

THRIVE’s first mpox case tested positive on July 8^th^, 2022 and received tecovirimat beginning July 15^th^, and AYAC’s first case was on September 13^th^. Swabs of pox lesions were initially tested at the Maryland Department of Health (MDH) for qualitative orthopoxvirus PCR and sent on to the Centers for Disease Control and Prevention (CDC) to exclude smallpox via qualitative variola PCR. Testing later moved to local laboratories for either qualitative orthopoxvirus PCR assays or eventually, mpox specific PCR. THRIVE was able to stock tecovirimat onsite through a partnership with the MDH starting July 26^th^. Jynneos mpox and smallpox vaccination was obtained through the Baltimore City Health Department and started being administered at both sites in October. Approximately 400 and 40 patients at THRIVE and AYAC belonging to groups disproportionally affected by mpox–GBMSM and transgender women–were called and offered vaccination. Patients were also offered vaccination through routine appointments at both sites.

### Study design

A PubMed search of human subjects publications including the terms mpox or monkeypox and either survey or experience or pain or stigma was conducted to identify any similar studies. Each was screened for relevance and for pertinent citations to review, and utilized in development of the survey.

Elements of the original 40-item HIV stigma scale [14] and abbreviated 12-item scale [15] were modified to develop a stigma survey for mpox. All 12 items from the abbreviated scale were utilized; this includes three questions each in categories ‘personalized stigma’, ‘disclosure concerns’, ‘concern about public attitudes’ and ‘negative self-image’. Six additional questions were taken from the 40-item scale, which were felt to have potential relevance to the mpox experience. The 19 modified stigma questions were sorted into the categories defined above, based on the highest item-factor correlation result from the initial stigma scale validation [14]. Five novel questions related to stigma were added and categorized by the authors.

Two open-ended questions regarding experience with the healthcare system were included: 1) *Can you comment further on your experience with the healthcare system*, *including the [THRIVE or AYAC] clinic*, and 2) *What aspects of testing*, *treatment*, *or follow up care helped or hindered your recovery*?

The survey also inquired regarding total number of mpox lesion, body sites involved, maximum pain level (1–10 numerical pain scale), pain management used, and effect of pain medications. Basic demographics, sex assigned at birth, current gender identity, gender(s) of sexual partners, types of sexual activity, route of mpox exposure, and HIV status were included in the survey.

Patients who were evaluated, tested, and/or treated for mpox at THRIVE or AYAC and tested positive for orthopoxvirus by PCR were included. Patients were contacted via telephone between October 2, 2022 and January 28, 2023 at numbers listed in their medical records, no earlier than 3-weeks following the date of diagnosis. Patients were deemed unreachable if they could not be contacted at all available numbers thrice. Patients reached were read study information and offered the opportunity to participate or decline. Verbal informed consent was obtained. There was no renumeration for participants.

This study was approved by the Institutional Review Board of the University of Maryland, Baltimore, protocol # HP-00103320. This study does not require patient consent.

This survey was created and conducted prior to the change from “monkeypox” to “mpox”. Therefore, the term ‘monkeypox’ is used when referring to survey questions, as this is how they were asked at the time. Otherwise the updated ‘mpox’ is used.

## Results

Thirty and two adult patients tested positive for mpox and received some or all of their mpox care at THRIVE or AYAC, respectively. Two died (one of severe mpox), two declined to participate in the phone survey, and four could not be reached. Surveys were completed for 24 patients. No patients at THRIVE or AYAC had previously been vaccinated with Jynneos.

Survey respondents had an average age of 37 (range 20–62), were cis-males (100%), mostly African American (88%) and non-Hispanic (96%), and living with HIV (79%). 96% reported that their sexual partners were male, and five of these men also had female partners. They reported engaging in anal receptive (83%), anal insertive (88%), oral receptive (88%), oral insertive (96%), and vaginal insertive (25%) sex, though when asked about the presumed route of inoculation only 8 of 24 mentioned sexual or close contact with unclothed people.

**Fig 1:**
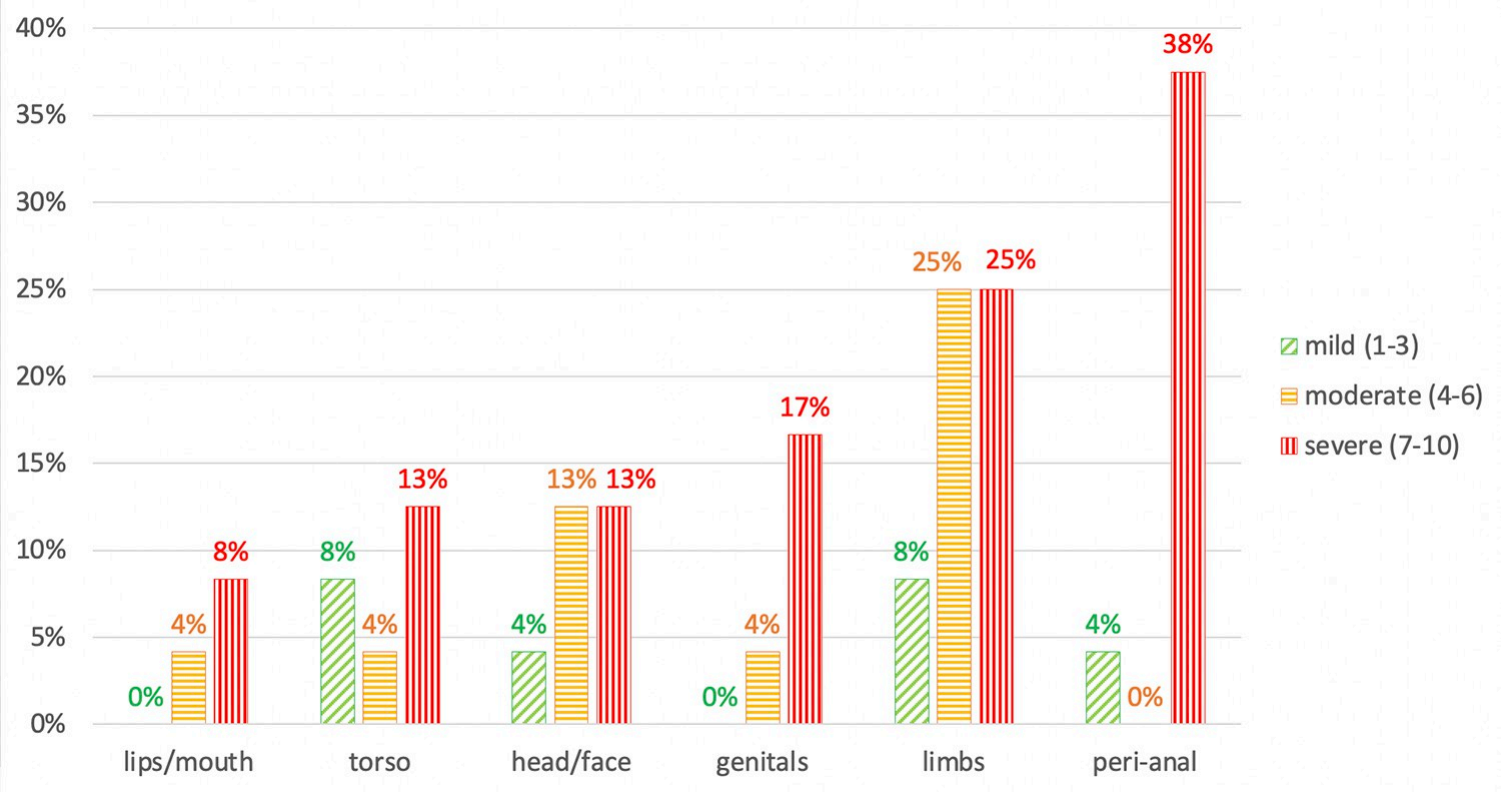
Percentage of patients reporting pain by body site and intensity.

The number of lesions was variable, with patients reporting <10 (46%), 10–100 (33%), or over 100 (21%), and 71% reported that lesion(s) were visible when wearing regular attire. Most (79%) had pain, with the most common sites being limbs (58%), perianal/anal (42%), head/face (29%), torso (25%), and genitals (21%). Perianal/anal pain was most commonly rated as severe (pain score of 7–10), with five patients rating the pain at a 10/10, four at a 9/10, and one at a 3/10 (Fig 1). Of 4 patients who did not report receptive anal intercourse (RAI), none had anal pain. Of those reporting RAI (n = 20), half had anal pain, which was reported as severe in 90%.

**Fig 2:**
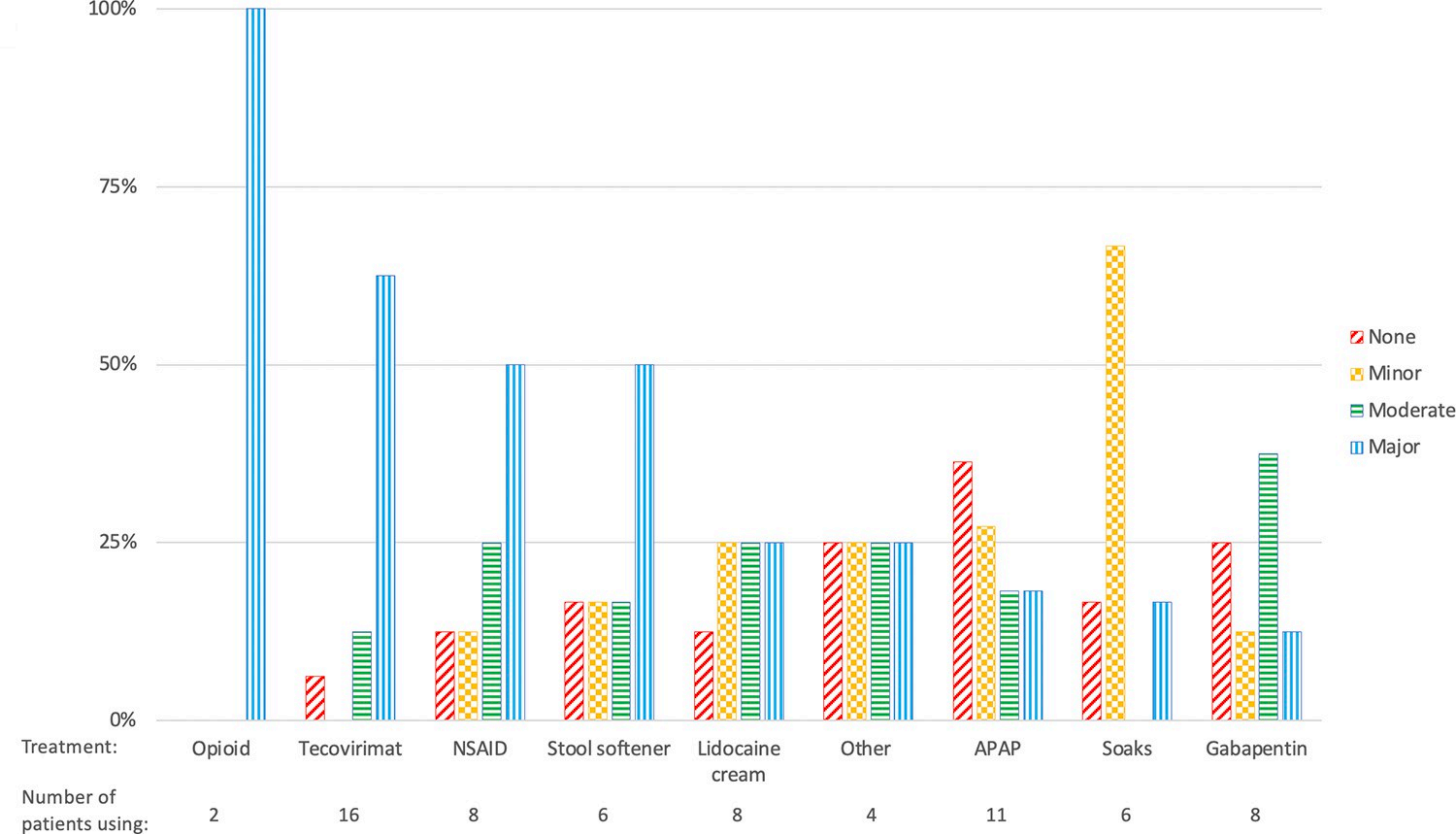
Pain treatment use and reported efficacy. Other treatments included 1 each: tetracycline, lotion, cocoa butter, and bleach baths.

Respondents reported major pain relief with opioids (2 of 2) and tecovirimat (10 of 16). Four reported moderate relief with tecovirimat, and two reported no improvement. Half reported major relief with non-steroidal anti-inflammatory drugs (NSAIDs) and stool softeners. Utility of lidocaine cream, acetaminophen, soaks, gabapentin, and other treatments had mixed responses, but for each, the majority of patients reported at least minor relief (Fig 2).

**Fig 3:**
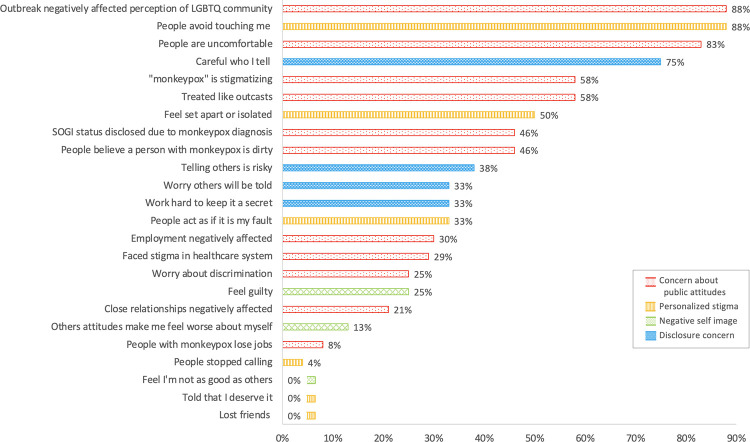
Responses to stigma question by question domain. Question stems are abbreviated; see Table 1 for full question text. LGBTQ: Lesbian, gay, bisexual, transgender, queer; SOGI: Sexual orientation and gender identity.

All patient answered ‘agree’ or ‘strongly agree’ to at least four stigma questions. The highest number of stigma questions answered affirmatively was 16 (n = 2). Mean and median were both 10 questions of 20. There were not significant differences in answers to each stigma question based on HIV status, whether lesions were visible, presence of any pain or anogenital pain, or receipt of tecovirimat. (Table 1) Patients had strongest agreement with questions in the domains of public attitudes and disclosure concerns (45% agree or strongly agree on average for both). Lower scores were seen for questions related to negative self-image (13%), and personalized stigma (29%). Several questions categorized as ‘personalized stigma’ could also have been answered affirmatively due to the visible and contagious nature of mpox infection rather than stigma; when excluded, the average affirmative response for personalized stigma questions dropped to 9%. (Fig 3)

**Table 1:** Patients answering ‘agree’ or ‘strongly agree’ to stigma questions.

	Total	HIV+	Visible lesions	Pain, any	Pain, anogenital	Received tecovirimat
	n = 24	n = 19	n = 17	n = 19	n = 12	n = 16
**Personalized stigma**						
People avoid touching me if they know I have monkeypox*^†^	21 (88%)	16 (84%)	14 (82%)	17 (89%)	11 (92%)	14 (88%)
People I care about stopped calling after learning I have or had monkeypox *^†^	1 (4%)	1 (5%)	1 (6%)	1 (5%)	1 (8%)	1 (6%)
I have lost friends by telling them I have or had monkeypox*^†^	0 (0%)	0 (0%)	0 (0%)	0 (0%)	0 (0%)	0 (0%)
Since learning I have monkeypox, I feel or felt set apart and isolated from the rest of the world ^†^	12 (50%)	9 (47%)	9 (53%)	10 (53%)	7 (58%)	9 (56%)
People have told me that getting monkeypox is what I deserve for how I have lived my life ^†^	0 (0%)	0 (0%)	0 (0%)	0 (0%)	0 (0%)	0 (0%)
Some people act as though it is my fault I have or had monkeypox ^†^	8 (33%)	5 (26%)	5 (29%)	8 (42%)	6 (50%)	6 (38%)
**Negative self-image**						
I feel guilty because I got monkeypox *^†^	6 (25%)	5 (26%)	2 (12%)	4 (21%)	2 (17%)	4 (25%)
People’s attitudes about monkeypox make me feel worse about myself *^†^	3 (13%)	1 (5%)	2 (12%)	3 (16%)	2 (17%)	2 (13%)
I feel I’m not as good as others because I have or had monkeypox *^†^	0 (0%)	0 (0%)	0 (0%)	0 (0%)	0 (0%)	0 (0%)
**Concerns about public attitudes**						
People with monkeypox are treated like outcasts *^†^	14 (58%)	11 (58%)	10 (59%)	12 (63%)	8 (67%)	11 (69%)
Most people believe that a person who has monkeypox is dirty *^†^	11 (46%)	8 (42%)	8 (47%)	10 (53%)	6 (50%)	8 (50%)
Most are uncomfortable around someone with monkeypox *^†^	20 (83%)	15 (79%)	14 (82%)	17 (89%)	10 (83%)	12 (75%)
People with monkeypox lose jobs when employers find out ^†^	2 (8%)	2 (11%)	2 (12%)	2 (11%)	1 (8%)	1 (6%)
Since learning I have monkeypox, I worry or worried about people discriminating against me ^†^	6 (25%)	4 (21%)	5 (29%)	6 (32%)	6 (50%)	6 (38%)
My employment was negatively affected by having monkeypox	8 (30%)	6 (32%)	5 (29%)	6 (32%)	3 (25%)	5 (31%)
Close relationships were negatively affected by having monkeypox	5 (21%)	4 (21%)	2 (12%)	4 (21%)	2 (17%)	3 (19%)
I believe that my sexual orientation or gender identity was disclosed by the fact that I have monkeypox	11 (46%)	7 (37%)	9 (53%)	10 (53%)	6 (50%)	8 (50%)
I believe that the current monkeypox outbreak has negatively affected perceptions of the LGBTQ community	21 (88%)	18 (95%)	16 (94%)	16 (84%)	11 (92%)	15 (94%)
I believe that the name ‘monkeypox’ is stigmatizing	14 (58%)	12 (63%)	13 (76%)	11 (58%)	6 (50%)	10 (63%)
I faced stigma in the healthcare system due to monkeypox	7 (29%)	5 (26%)	5 (29%)	5 (26%)	3 (25%)	5 (31%)
**Disclosure concerns**						
Telling someone I have or had monkeypox is risky *^†^	9 (38%)	7 (37%)	7 (41%)	7 (37%)	5 (42%)	7 (44%)
I work hard to keep my monkeypox a secret *^†^	8 (33%)	7 (37%)	5 (29%)	6 (32%)	5 (42%)	7 (44%)
I am very careful whom I tell that I have or had monkeypox*^†^	18 (75%)	14 (74%)	12 (71%)	14 (74%)	9 (75%)	14 (88%)
I worry or worried people who know or knew I have monkeypox will tell others ^†^	8 (33%)	5 (26%)	7 (41%)	6 (32%)	5 (42%)	7 (44%)

*: Adapted from HIV Stigma Scale; also included in Abbreviated HIV Stigma Scale

†: Adapted from HIV Stigma Scale

Representative answers to two open-ended questions about health care experiences are included in Table 2 and demonstrated satisfaction and gratitude for services provided at THRIVE and AYAC. Healthcare experiences outside of or prior to engagement with THRIVE and AYAC were universally negative, citing uncertainty in how to get care, delays, and lack of tecovirimat access.

**Table 2:** Example responses to ‘healthcare experience’ questions.

THRIVE was pretty awesome. They cater to the LGBTQ community, so it was pretty easy.
AYAC diagnosed me ASAP and gave me medicine for the symptoms while waiting for my results. AYAC helped me get into treatment. THRIVE provided treatment which was amazing and helped right away.
They helped me out at THRIVE. The ER didn’t know what was going on.
I’m very happy with THRIVE. It was quite wonderful. Tried [other clinic] first and they refused to give me treatment.
It was challenging to figure out where to get help once I got diagnosed, but once I figured that out everything was easy. [THRIVE]
Wonderful. Overall the experience was great, especially with my nurse and provider. They were very attentive, contacted me every other day, and made sure I had follow-up. Social work also helped make sure I could get food to my house. [THRIVE]
THRIVE was great, excellent. I appreciate everybody who cared for me. They were very concerned and advocated for me. They put me first.
The process for testing was slow, super delayed. I was hospitalized for another reason when a nurse noticed one spot. When the second spot appeared, I was put in isolation and had to wait 5 days for the result to return before I could go home.
Very impressed with my doctor who immediately recognized I had monkeypox when I thought it was a bug bite. Very impressed with the coordination between my provider and the health department. Provider was engaged. I got daily telephone calls to keep me in the loop and check up on me. Had no problems. [THRIVE]
Everyone was very nice and very supportive and were there when I had any questions or concerns. They called back real quick when I was first worried that I might have monkeypox. [THRIVE]
It was kind of off-putting for the initial diagnosis in the ER. I was told they didn’t have TPOXX available, and that I’d have to let the process run its course naturally, so I was out of work for 6 weeks until everything healed.
AYAC did a wonderful job treating and staying on top of my symptoms. I had a very mild case and no pain, but they called a bunch of times to check on me.
THRIVE is great. Everyone is always upbeat and hospitable. The doctor was very honest about the infection, what it would do and what it wouldn’t do. They called and checked on me, even gave their personal phone number. Really good.
The telemed video visits helped because I could show where the spots were and how they were improving. [THRIVE]
Once I got on TPOXX that set me on the path to feeling better. That was a major turning point. [THRIVE]

LGBTQ: Lesbian, gay, bisexual, transgender, queer

AYAC: Adolescent and young adults clinic

ASAP: As soon as possible

ER: Emergency room

## Discussion

This is the first qualitative or quantitative description of mpox patients’ experience with stigma, pain, and health care systems during the 2022 outbreak. Our findings confirm prior reports of severe pain and concerns over potential stigma and general poor experience with health systems, but also demonstrate which pain management modalities were most effective, and that it is possible to make patients feel welcomed and cared for.

Participants in this study were already members of historically stigmatized groups–mostly African American, living with HIV, and GBMSM. While a high rate of HIV infection among mpox patients is recognized, the higher rate in this series compared to prior reports is due to studied clinics primarily servicing people living with HIV (PLWH).

Interestingly, the stigma domains that patients most consistently reported agreement with were related to public attitudes and disclosure concerns, but they had lower scores for negative self-image and personalized stigma. UNAIDS released a statement early in the 2022 mpox outbreak “that some public reporting and commentary on Monkeypox has used language and imagery, particularly portrayals of LGBTI and African people, that reinforce homophobic and racist stereotypes and exacerbate stigma [12]”. A machine learning analysis of over 300,000 English-language twitter posts early in the outbreak revealed that 11.3% fit the theme of ‘stigma towards minority communities’ [16]; the three example tweets cited each expressed concern that stigma *could* result. None were directly stigmatizing or discriminatory. That mpox and messaging about it may be stigmatizing is a clear concern to the general population and to the medical and public health communities and was verified as a concern to patients here. It is of some relief that the patients surveyed didn’t have the same degree of internally directed negativity. The highest scored question regarding stigma (tied with “people avoid touching me…”) was “I believe that the current monkeypox outbreak has negatively affected perceptions of the LGBTQ community”. About half felt that their sexual orientation or gender identity (SOGI) was disclosed by their mpox diagnosis. Overall, 58% agreed that the name ‘monkeypox’ is stigmatizing, and this rate appeared to be higher (76%) in those with visible lesions.

The only other study assessing anticipated mpox stigma surveyed black sexual minority men receiving mpox vaccination in Washington DC. 13.5%, 25.8%, 28.7%, and 31.5% agreed or strongly agreed that friends, family, the LGBTQ community, and sexual partners, respectively, would think less of them if they found out they contracted mpox [17]. Respondents agreed or strongly agreed that people would blame them (35.4%), assume sexual promiscuity (51.7%), or assume they were gay or bisexual (48.3%), though only 5.6% felt that mpox is a “gay disease”, suggesting a gap between their perception of the association of mpox and GBMSM identity (low) versus how they believe the public perceives this association (high) [17].

A limitation of using the HIV-stigma scale as a starting point for mpox stigma assessment in this study is the impact of visible and contagious lesions in mpox. Questions such as “people avoid touching me… “, “people with monkeypox are treated like outcasts”, “most people believe that a person who has monkeypox is dirty”, and “most are uncomfortable around someone with monkeypox” may be reflective of an appropriate caution regarding contracting the illness, rather than stigmatizing beliefs. Similarly, the question about feeling isolated is likely reflective of recommended quarantine periods. However, regardless of the exact reason for agreement with any of these questions, each would still connote a difficult or negative experience due to mpox.

Understanding the stigmatizing experience of mpox in general, overlaid with potentially race and SOGI-based stigma or discrimination is critical in developing a constructive public health response to this and similar future outbreaks. Debate over public health approaches that correctly target and inform those at highest risk without downplaying the risk for others or inadvertently increasing discrimination of SGM began early in the outbreak. UNAIDS and the CDC released guidance on messaging, recommending a focus on transmission behaviors and routes rather than groups at highest risk, and separate messaging goals for a general audience versus GBMSM communities [12, 18]. In addition to the obvious and important goal of avoiding contributing to or exacerbating stigma in already socially and medically marginalized populations, many have pointed out that stigma of illnesses and people with them undermines an effective outbreak response [12, 19]. Framing any illness as only or predominantly occurring in GBMSM will naturally lead to avoidance of care if there is danger associated with identifying as GBMSM. This was the case in a Thai man diagnosed with mpox who fled public health authorities following his diagnosis, and was ‘captured’ after a countrywide chase, and as reported “infected many others” [19].

The 2022 mpox outbreak is a continuation of an outbreak in Nigeria that started in 2017, and affected 226 people between 2017–2022 [20], yet garnered little international attention. Of note is the lack of mention of affected patients’ sexual practices or SOGI despite the first case series including 78% men, 23% PLWH, 68% with a genital rash, and most cases occurring only in “young men, ages 20–40” [20, 21]. This may have been intentional, as same-sex relations are illegal in Nigeria since the “Same Sex Marriage (Prohibition) Act” was signed into law in 2014, punishable by up to 10-years imprisonment [22]. Thus, correlating mpox infection with LGBTQ identity also poses a potential physical danger depending on local laws and cultural norms.

Similarly, the framing of HIV initially as a ‘gay disease’, including terms such as “gay related immune defense disorder,” or “gay-related immune deficiency”, has undoubtedly resulted in countless cases of missed or delayed diagnoses and persistent stigma that continues to threaten HIV epidemic control 40-years later [23].

While the importance and benefit of crafting public health policy and messaging to avoid ever stigmatizing any group cannot be understated, it is also worth noting that the 2022 mpox outbreak ended fairly swiftly which may be in part due to public understanding of mpox as a disease primarily affected GBMSM. An August 2022 survey of MSM showed that 90.4% did not ever think they had mpox and 97.6% had not been tested, but 22.9% had already received mpox vaccine and 56.0% had changed their sexual behavior due to the outbreak [24]. Another August survey of 824 MSM found that number of sex partners, one-time sexual encounters, and sex with partners met on dating apps or at sex venues was reduced in 48%, 50%, and 50% since learning about the mpox outbreak [25]. One must ask–if they had not been appropriately informed of their inclusion in a group disproportionally affected, would GBMSM have adjusted sexual behaviors and been vaccinated, this curtailing the outbreak?

Equally important during this outbreak has been the need to acknowledge patients experiences of severe pain, particularly proctitis, which was prominent [3–8] and not seen in ‘classic’ mpox cases, and manage it effectively. Anogenital lesions were reported in as many as 94% of patients [5], often causing severe pain, and uncontrolled pain was a top reason for hospitalization [5, 7, 26]. The high rates and severity of pain from both cutaneous and anal/peri-anal lesions is confirmed in this study, and warrants real attention, particularly in a disease that is already stigmatizing and isolating.

CDC guidance on pain management recommended acetaminophen, NSAIDs, topical steroids, topical anesthetics, and gabapentin or opioids as necessary, in addition to stool softeners and sitz baths for those with painful proctitis [27]. Use of topical lidocaine [3, 28, 29], rectal suppositories [3], oral laxatives [3], paracetamol [3, 29], ibuprofen [3, 28, 29], methimazole [28], tramadol [28], opioids [3], tecovirimat [26], and photodynamic therapy for cutaneous lesions [30] have been reported in case reports and series, though without analysis of efficacy. While the utility of tecovirimat for pain and lesion resolution is currently being evaluated in a clinical trial [31], early indications for its use per the CDC did include “other anatomic areas where monkeypox virus infection might constitute a special hazard (e.g., the genitals or anus)” and “people with immunocompromising conditions (e.g., HIV/AIDS…)” [11]. These indications did provide physicians with an opportunity to utilize this antiviral in people living with HIV (depending on interpretation of above), and those with anogenital involvement which is clearly associated with severe pain; this is presumed to be the impetus behind a September 15, 2022 CDC clinician letter warning against ‘indiscriminate use’ [11] of tecovirimat. Patients in this study did in fact report highest rates of pain relief from opioids and tecovirimat, with mixed efficacy of other modalities. The outcomes of the STOMP trial [11] will hopefully lend additional outcomes data to support the use of tecovirimat for relief of severe mpox pain.

The THRIVE and AYAC clinics were fortunate to have the capacity, expertise, and relationships with public health entities that allowed for our clinics to become referral sites and see a majority of mpox patients in the area. Patient comments related to health care experience provide proof that people can have a positive experience if the correct resources are in place, and also that less positive experiences arise at sites without the resources and expertise to address their needs. HIV and STI clinics are well positioned to respond to similar outbreaks and emerging infections, especially those affecting marginalized groups that are already in their care or that view these clinics as ‘safe-spaces’.

The ability to statistically analyze responses was hampered by the small sample size of this study. The relatively small size of this outbreak, despite its global scale, limits much detailed study of the disease at a local level. Multi-institution data aggregation is warranted to generate additional actionable findings.

## Conclusions

Patients diagnosed with mpox experienced significant pain and stigma and had positive health care experiences at two primarily HIV care/prevention clinics offering mpox care, but negative experiences elsewhere. Opioids and tecovirimat appear to be the most effective available pain management options, though larger studies are warranted. HIV/STI clinics catering to already stigmatized populations can successfully pivot to expediently offer services related to care of people experiencing emerging infections.

## Supporting information

S1 Checklist Human participants research checklist. (DOCX)

S1 Data (XLSX)
